# The role of ocelli in cockroach optomotor performance

**DOI:** 10.1007/s00359-017-1235-z

**Published:** 2017-11-30

**Authors:** Anna Honkanen, Paulus Saari, Jouni Takalo, Kyösti Heimonen, Matti Weckström

**Affiliations:** 1 Nano and Molecular Systems Research Unit, University of Oulu, P.O. Box 3000, 90014 Oulu, Finland; 2 Present Address: Vision Group, Department of Biology, Lund University, 223 62 Lund, Sweden; 3 Present Address: Centre for Cognition in Small Brains, Department of Biomedical Science, University of Sheffield, Sheffield, S10 2TN UK

**Keywords:** Optomotor reaction, Behaviour, Ocelli, Cuticular transmission, Virtual reality

## Abstract

Insect ocelli are relatively simple eyes that have been assigned various functions not related to pictorial vision. In some species they function as sensors of ambient light intensity, from which information is relayed to various parts of the nervous system, e.g., for the control of circadian rhythms. In this work we have investigated the possibility that the ocellar light stimulation changes the properties of the optomotor performance of the cockroach *Periplaneta americana*. We used a virtual reality environment where a panoramic moving image is presented to the cockroach while its movements are recorded with a trackball. Previously we have shown that the optomotor reaction of the cockroach persists down to the intensity of moonless night sky, equivalent to less than 0.1 photons/s being absorbed by each compound eye photoreceptor. By occluding the compound eyes, the ocelli, or both, we show that the ocellar stimulation can change the intensity dependence of the optomotor reaction, indicating involvement of the ocellar visual system in the information processing of movement. We also measured the cuticular transmission, which, although relatively large, is unlikely to contribute profoundly to ocellar function, but may be significant in determining the mean activity level of completely blinded cockroaches.

## Introduction

The compound eye is the main visual organ in an adult insect, but many species possess one, two, or three ocelli (singular: ocellus) (Krapp 2009; Leschen and Beutel 2004; Mizunami 1995b) in addition. Unlike the multifaceted compound eye, an ocellus has only one lens over a disorderly packed retina. In exceptional cases apparently functional ocelli can exist without any external lens (Eaton 1971; Pappas and Eaton 1977). The retinula cells making up the photoreceptive rhabdoms typically converge upon a few large ocellar second-order neurons (L-neurons) (Mizunami 1995b). Sensitivity of ocelli can be enhanced with a tapetal layer or the formation of palisade reflecting stray photons back to the ocellar retina (Goodman 1970; Mizunami 1995b). The focal point of a typical ocellar lens lies behind the retina and the ocellus is thus strongly underfocused and unable to form an image. Instead, coupled with the high convergence of the photoreceptors to the second-order neurons, ocelli may be able to detect subtle changes of ambient light intensity, or contribute to flight stability by detecting rapid changes in the pitch and yaw orientation (Krapp 2009). However, some insects do have ocelli that can focus light on the retina, typically to facilitate horizon detection (Stange et al. 2002). Ocelli can also be used in navigation for detecting the e-vector direction of polarised light (Fent and Wehner 1985; Mote and Wehner 1980; Ribi et al. 2011; Taylor et al. 2016).

The American cockroach, *Periplaneta americana* (L.), has two white ocelli upon the frons. Their lenses are flat or somewhat concave and remarkably large, 0.7 mm in diameter, giving them an extremely wide receptive field (Weber and Renner 1976; Mizunami 1995b). The retina contains about 10 000 green-sensitive photoreceptors that converge onto only four second-order L-neurons in the ocellar plexus—the largest convergence ratio known for any insect (Mizunami 1995d; Weber and Renner 1976). Synapses between photoreceptors and feedback synapses from L-neurons onto photoreceptors are rare (Toh and Sagara 1984). The L-neuron axons leave the ocellus and enter the ocellar tract neuropil of the brain via the ocellar nerve. Peculiarly, none of the L-neurons in the cockroach brain project to the posterior slope but instead synapse exclusively in the ocellar tract (Mizunami et al. 1982; Mizunami 1995b).

The synapsing pattern of an ocellar system reflects the behavioural tasks guided by the ocellar information. The three known synapsing types are the fast but not very sensitive bisynaptic, the slow and sensitive trisynaptic, and the fast and sensitive intermediate system (Mizunami 1995b). In bisynaptic system the photoreceptor signals are conveyed to target neuropils directly by a relatively large number of second-order neurons. The American cockroach has the trisynaptic system where photoreceptor signals first converge onto just four second-order neurons which in turn make connections with a large number of third-order neurons, passing information to the target neuropils. All the three optic neuropils (lamina, medulla and lobula) of the cockroach receive ocellar inputs (Mizunami 1995c). A connection between the ocellar nerve and the lamina has also been found in the cricket, and is suggested to control the compound eye sensitivity (Rence et al. 1988).

Behavioural experiments with occluded ocelli or compound eyes, and studies where the ocelli are stimulated, have revealed some possible functions of the ocelli in various taxa. These include orientation and phototaxis (Hu and Stark 1980; Lazzari et al. 1998; Wehrhahn 1984), navigation and polarisation vision (Fent and Wehner 1985; Wellington 1953, 1974), circadian timing of activity periods in (Eaton et al. 1983; Rence et al. 1988; Wunderer and De Kramer 1989), and flight control (Parsons et al. 2006; Taylor 1981; Van Kleef et al. 2008). The ocelli of honeybees are even suggested to improve colour constancy in changing ambient illumination (Garcia et al. 2017).

In spite of large number of experimental work in other species, so far there is not much behavioural evidence for the actual role of ocelli in cockroaches. It is known that ocelli are not needed for circadian photoentrainment (Roberts 1965), and that ocellar occlusion impedes the shade response at low-light levels, indicating a role in light detection and modulation of compound eye signals (Okada and Toh 1998). In bright light the cockroach *Blaberus discoidalis*, confronted with a shelf-like obstacle, prefers to crawl under it, whereas in darkness as well as in bright light with its ocelli and compound eyes—or just the ocelli—covered, it makes a random choice between climbing over and tunnelling under the obstacle. Covering only the compound eyes results in tunnelling. This observation suggests a role for the ocelli in determining whether the animal concentrates on foraging activity or shelter finding (Harley et al. 2009). In the present work, we investigated the effects of ocellar or compound eye occlusion on the optomotor performance in the American cockroach, to find out if the behaviourally determined optomotor response is controlled or tuned by the ocellar light stimulation.

## Materials and methods

### Behavioural experiments and data analysis

The behavioural experiments were carried out in a panoramic virtual reality (VR) system for insects, as described previously (Takalo et al. 2012; Honkanen et al. 2014). Briefly, the system consists of a projector (DepthQ^®^, Lightspeed Design Inc., USA) equipped with a fisheye lens via which stimuli are produced onto the inside of a spherical projection surface covering 270° in horizontal plane. The spectrum of the projected image contains wavelengths between 400 and 700 nm (Takalo et al. 2012), i.e., it excites almost exclusively the green receptors (Zhukovskaya et al. 2017). An air-suspended lightweight trackball (diameter 93 mm, weight 4.8 g) is placed inside the projection sphere, so that the compound eyes of an insect walking on the trackball are at the equator of the sphere. Movements of the animal rotate the trackball, and these rotations are detected by a pair of optical computer mice and subsequently recorded on a computer.

The stimulus is a horizontally rotating grating of vertical black and white bars with an angular period of 60° and temporal frequencies of 0.1, 0.4, 2.4, 4, 6, 10, 12, 15, and 18 Hz. The unattenuated light intensity inside the sphere during stimulation is 500 lx (5 × 10^14^ photons/cm^2^/s), and lower intensities are achieved using − 2, − 4, and − 5 decade neutral density filters (NE Series, Thorlabs, Newton, NJ, USA) in front of the projector lens. The Michelson contrast of the stimulus pattern remained 0.33 at all light intensities. Each stimulus started with a 30-s control during which the grating pattern was visible but did not move. Control was followed by a 30-s rotation to randomly chosen direction (either clockwise or anticlockwise); then, a 15-s control followed by a 30-s rotation to the opposite direction from the first rotation. The total stimulus duration was thus 105 s, after which there was a 20-s complete darkness before the next stimulus started.

Data were analysed with MATLAB R2013b (The MathWorks, USA) and statistical tests run with OriginPro 8.6 (OriginLab Corporation, USA). The response strengths were calculated as in Honkanen et al. (2014): response strength = 2(Angle_follow_/Angle_total_) − 1, where Angle_total_ is the total angular distance covered by the cockroach during the rotating stimulus or control (Angle_total_ = Angle_follow_ + Angle_wrong_), and Angle_follow_ is the angular distance moved in the direction of the stimulus.

For estimating the walking activity in Table 1, the maximum velocities and total distances walked were directly readable from the trackball outputs. The average velocities and the number of full rotations were calculated from the total distances by dividing them with the trial duration and 360°, respectively. In Table 2, the inactivities were calculated as the percentage, out of all trials of each group, of those trials in which the animal walked for less than one full rotation during the whole time.

### Animals and preparation

Adult male American cockroaches (*Periplaneta americana*) from a commercial supplier (Blades Biological Ltd., Cowden, Kent, UK) were used. In the laboratory, they were housed separately at room temperature (about 20 °C) with 12:12 light:dark rhythm and given food and water ad libitum. All the experiments were performed during the dark phase of the rhythm, although some experiments extended into the beginning of the light phase.

The animals were cold-anaesthetised in a refrigerator and preparations were done on an ice brick. The ocelli were covered either by fixing a piece of aluminium foil on the lens with glue, or painting over the lens first with red nail polish and, after it had dried, with grey furniture paint. The compound eyes were always covered with the combination of nail polish and furniture paint. The eye coverage was checked before the beginning of each experiment and immediately after it. Only data from experiments where the ocelli and/or compound eyes had remained completely covered for the duration of the entire experiment were accepted for analysis.

After covering the eyes, a holder made of metal wire was fixed onto the pronotum of the cockroach with a mixture of beeswax and resin. Full recovery from the cold anaesthesia was required before starting the experiment, and judged from the recovery of locomotion and antennal movements. The animal was placed onto the trackball in a posture as natural as possible, so that it was able to perform walking movements and turn its head and abdomen, but not touch the trackball with its mouthparts. The cockroaches were able to touch the trackball with their antennae but such contacts were rare, and the absence of chemosensory and tactile cues kept the animals motivated to walk up to hours at a time. The spherical projection surface allowed the stimulation of both the compound eyes [nearly full spherical field of view (Butler 1973)] and the ocelli, which were pointed mainly towards the lower frontal hemisphere of the projection surface.

In experiments of the spectral properties of the cuticle, the left compound eye with the ocellus and the base of the antenna was cut off with a razor blade after CO_2_ anaesthesia, decapitation, and removal of antennae. The subcutaneous tissue behind measuring areas, including the ocellar tissue, was cleaned out and the sample was cut into pieces of measurable size. For absorption recordings, each sample piece was carefully placed the outer surface up in a drop of insect Ringer’s solution (Salmela et al. 2012; bath solution in the patch-clamp recordings) on the cover glass. The upper cover glass was not used due to the short measuring time and differences in sample thickness, but the hydration of bath solution was controlled throughout the experiment.

### Spectrophotometrical recordings and data analysis

**Fig. 1:**
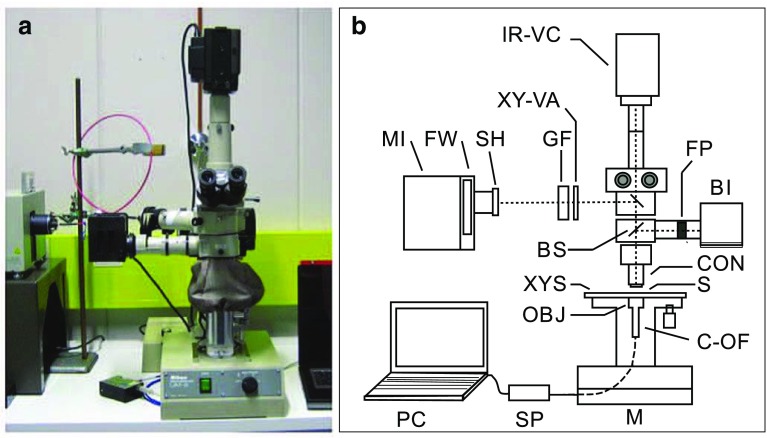
Experimental setup for cuticle measurements (**a**) and its schematic principle (**b**). *MI* measuring illuminator, *FW* filter wheel, *SH* shutter, *GF* grey filter (optional), *XY-VA* X-Y variable circular zero aperture, *IR-VC* infrared sensitised video camera (connected to a monitor; not shown), *BS* beam splitter, *BI* background illuminator, *FP* filter pack, *CON* condenser, *XYS* xy-stage, *S* specimen, *OBJ* objective, *C-OF* collimator-optic fibre system, *M* microscope, *SP* spectrometer, *PC* computer with the SpectraSuite software

Absorption measurements of head capsule cuticle were conducted in an experimental setup (Fig. 1a, b) constructed around a fluorescence microscope (Nikon Measurescope, UM-2, Japan). In brief, the measuring beam, provided by a 75 W xenon arc lamp (Oriel Instruments, USA) was fed by a quartz optic fibre cable and a collimating lens (Oriel Instruments, USA) into the horizontal optical path of the microscope. The beam size was controlled by a variable circular aperture and focused onto the specimen level by microscope optics and an objective (Nikon, 40×/NA 0.5) working as a condenser. The background illumination was produced by a 100 W long-pass filtered (*f*_c_ = 760 nm) halogen lamp, and to control the sample, both oculars and infrared sensitised video camera could be used. The objective (Reichert 95×/NA 0.95 or Nikon 60×/NA 0.70) followed by a quartz collimator-optic fibre combination (Ocean Optics Inc., USA) gathered the transmitting beam, and introduced it onto the detector of a miniature UV–Vis spectrometer (USB4000, Ocean Optics Inc., USA).

Recordings were performed at room temperature. First, the objective was focused onto the specimen level, and then, the measurement position was found and focused onto. The accurate position was determined, so that the shape of sample spectrum and the reflection of the measurement beam, mainly due to high refractive index and uneven surface of the material, respectively, indicated perpendicularity to the beam. To measure the spectral absorbance of cuticle and antenna structures, two spectra were compared. First, a reference spectrum was registered by placing the measurement beam near the sample in a clear tissue-free area, and subsequently, a sample spectrum was recorded. Background level spectrum was subtracted automatically from every spectrum. Spectral absorbance was then calculated as 10-base logarithm of the ratio between the reference and sample intensity spectrum. For every sample spectrum, 20–100 successively registered spectra with ca. 20-point running ‘boxcar’ smoothing were averaged. The measuring data were stored in Excel (2010, Microsoft, USA) data spreadsheets and analysed by MATLAB R2013b (The MathWorks, USA). The averaged absorbance spectrum for each target position was calculated based on their transmittance spectra and the error bars (± SD) directly from the absorbance values. Illustration was done using OriginPro 2015 (OriginLab Corporation, USA).

## Results

### Ocellar and compound eye occlusion

**Fig. 2:**
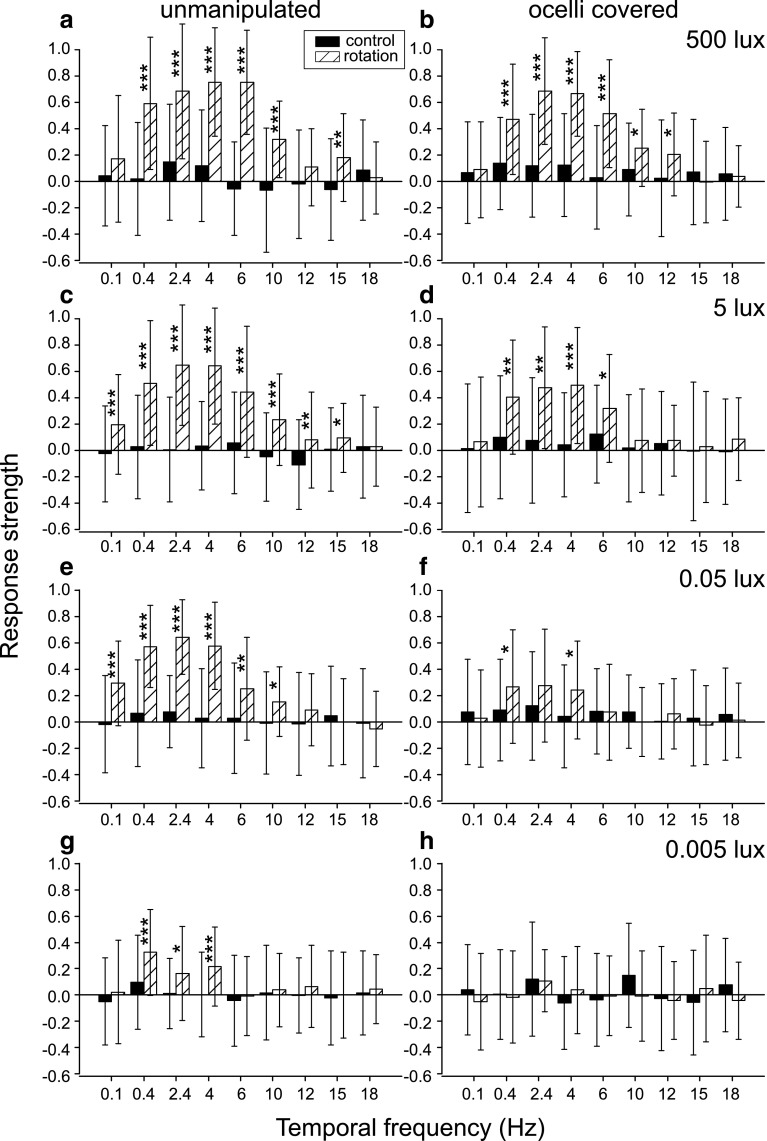
Optomotor response strengths ± SD to different temporal frequencies of the stimulus with 60° angular period. Data from the “unmanipulated” group are shown in the left column and data from the “ocelli covered” group on the right. Light intensities are shown in the upper right corner of each row. Solid bars represent the strength of the response during the stationary controls and hatched bars during rotating stimuli. Response strengths range between 1 for the strongest positive and − 1 for the strongest negative (anti-directional) response. The expected control level is zero. One, two, and three asterisks indicate significant differences between the control and rotation distributions at confidence levels of, respectively, 0.05, 0.01, and 0.001 (paired sample Wilcoxon signed-rank test). **a**–**d** Attenuation of the response strengths, and the narrowing of the frequency band that is able to elicit the optomotor response, with falling light levels. **e**–**h** When the ocelli are covered, the response strength attenuates, and the frequency band narrows, at higher light intensity levels than in unmanipulated cockroaches. Sample sizes were **a** *N* = 20 animals, *n* = 40 measurements; **b** *N* = 24, *n* = 78; **c, d** *N* = 23, *n* = 66 and **e–h** *N* = 20, *n* = 40. Data in **a**–**d** are from the data set in Honkanen et al. (2014)

Behavioural experiments with optomotor stimuli in the VR demonstrate that the ocelli affect the optomotor responses of the American cockroach. Figure 2 compares the optomotor responses of cockroaches in the control group (left column, “unmanipulated”) and cockroaches whose ocelli have been covered (right column, “ocelli covered”) in four different light intensities between 500 and 0.005 lx. At 500 lx (Fig. 2a, e) the responses of both groups look similar: the strongest responses are about 0.7 on the response strength scale (Honkanen et al. 2014), and stimulus frequencies of 0.1 and 18 Hz do not elicit any responses; neither does 15 Hz in the “ocelli covered” group.

Exposed to 5 lx light intensity (Fig. 2b, f), the strongest mean responses of the “ocelli covered” group reach only 0.5 on the response strength scale which is weaker than the top responses of the “unmanipulated” group at a hundred times lower light intensity of 0.05 lx The “ocelli covered” cockroaches are only able to respond to stimulus frequencies between 0.4 and 6 Hz compared to 0.1–15 Hz in the “unmanipulated” group.

0.05 lx intensity (Fig. 2c, g) produces hardly any significant responses in the “ocelli covered” group. In fact, the responses are, with the same stimulus frequencies, similar or even weaker than the responses of the “unmanipulated” animals at the ten times lower light intensity of 0.005 lx. In comparison, the responses of the “unmanipulated” group at this light intensity resemble the responses of the “ocelli covered” group at 500 lx only shifted towards the low frequencies.

At the lowest light intensity where the “unmanipulated” cockroaches are still able to respond to optomotor stimuli, 0.005 lx (Honkanen et al. 2014), the animals whose ocelli are covered do not react to the stimulus at all (Fig. 2d, h). The absolute behavioural threshold for the “ocelli covered” group is, therefore, at least at 1 decade higher light intensity than the threshold in cockroaches that can use all of their four eyes.

**Fig. 3:**
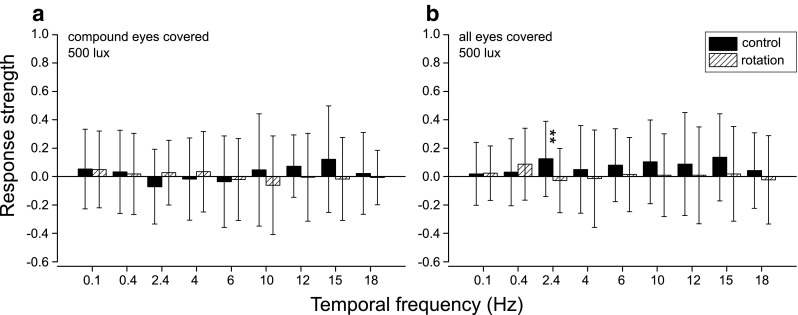
Response strengths of cockroaches whose compound eyes or all eyes have been covered at 500 lx. See Fig. 2 for the symbol keys. **a** No significant differences between control and rotation values were found. **b** Significant difference (*p* = 0.00256, paired sample Wilcoxon signed-rank test) between control and rotation values was found at 2.4 Hz, where the control value happens to be on the positive and the rotation value on the negative side. The responses in **a** and **b** are almost identical. Sample sizes were **a** *N* = 20 animals, *n* = 40 measurements; **b** *N* = 19, *n* = 38

For control, optomotor responses of two more treatment groups were tested: cockroaches whose compound eyes were covered, but ocelli left intact (Fig. 3a), and cockroaches who had both the compound eyes and ocelli covered (Fig. 3b). The responses of these groups do not differ from their controls even at the highest light level of 500 lx. They are identical to responses of the “ocelli covered” group at 0.005 lx (Fig. 2h) and “unmanipulated” group at 0.0005 lx (Honkanen et al. 2014), indicating that the animals cannot see the stimulus.

### Effect on general activity levels

**Fig. 4:**
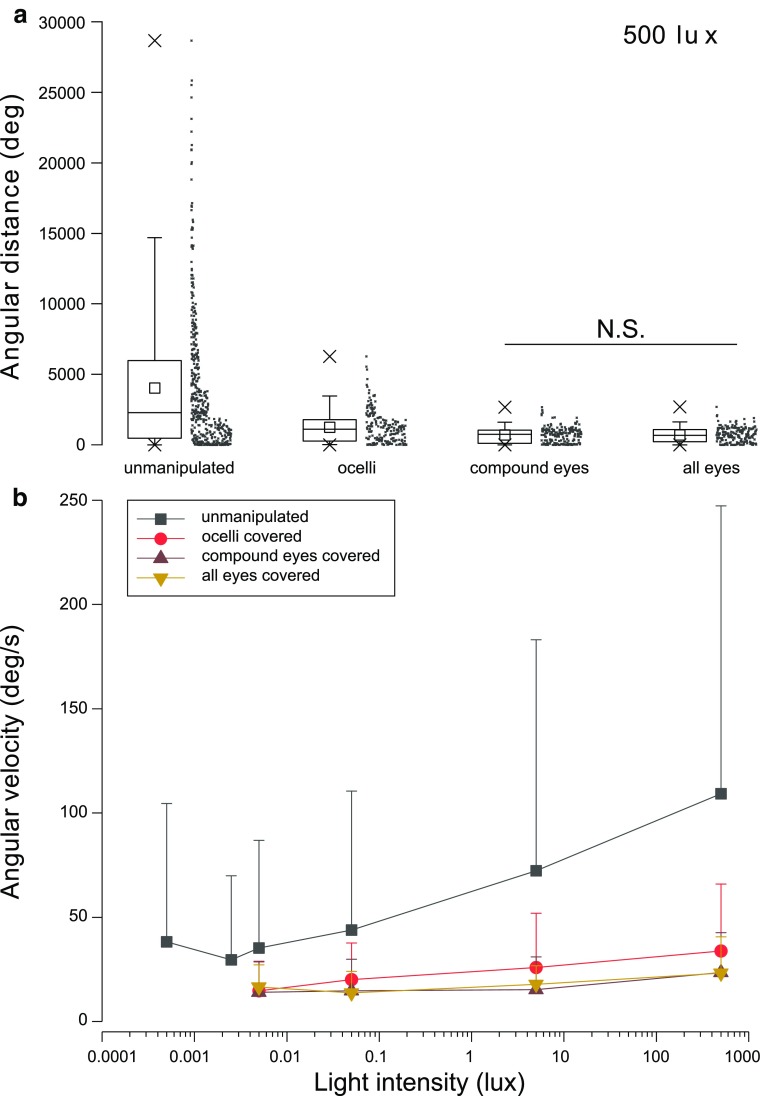
Total distances and average velocities walked by the cockroach during the 30-s stimulus rotation. **a** Boxplots and data point distributions of the total angular distances covered by the cockroaches during stimulus rotation. “Unmanipulated” is the condition where none of the eyes are covered. “Ocelli”, “compound eyes”, and “all eyes” denote which eyes are covered in the three treatment groups. The box plot shows the first and third quartiles and the median of the data; the square inside the box plot marks the mean; the “whiskers” of the plot are the 95th and 5th percentile; the maximum and minimum values of the data are marked with “X”. Pairwise differences between treatments were tested with two-sample Kolmogorov–Smirnov test. All other groups differ from each other significantly (*p* < 0.001) except “compound eyes” and “all eyes” (N.S.; *p* = 0.55). Data from experiments with 0.4–10 Hz stimuli are combined to produce the plots. Sample sizes are *n* = 462 (Unmanipulated); *n* = 200 (Ocelli and compound eyes); *n* = 190 (All eyes). **b** Average angular walking velocities ± SD of cockroaches with different eye manipulations (insert) across different light intensities. Each data set contains data from all nine temporal frequencies of the stimulus. The mean velocities of all the groups rise with rising light intensity. In pairwise comparisons, all data points of “unmanipulated” group differ from the other three groups (two-tailed two-sample Kolmogorov–Smirnov test *p* < 0.001) and all data points of “ocelli covered” group differ from the “compound eyes covered” group (*p* < 0.01). The averages of “compound eyes covered” and “all eyes covered” groups are not significantly different from each other at 500 lx (*p* = 0.09) and are closely identical but significantly different (*p* < 0.05) at all other light intensities. Sample sizes per data point *n* = 216–920 (unmanipulated); *n* = 360 (Ocelli and compound eyes covered), *n* = 90–342 (all eyes covered)

The general activity level of the cockroaches is affected by occlusion of the ocelli or compound eyes or both. Figure 4a demonstrates that at 500 lx, the total angular distance walked by the animals during stimulus rotation is significantly shorter in all three manipulation groups compared to the “unmanipulated” group (data are combined from experiments with 0.4–10 Hz stimuli which are most likely to produce optomotor responses; see also Fig. 2a, e). Medians are 2284.6° (unmanipulated), 1107.6° (ocelli covered), 757.3° (compound eyes covered), and 671.3° (all eyes covered). Covering the compound eyes has a similar effect on the total walking distance as covering all eyes (see also Fig. 3). Covering the ocelli causes intermediate walking activity, although the median and mean of these data are closer to those of the other two manipulation groups than to the “unmanipulated” group (see also Table 1 calculated with data containing all stimulus frequencies). Comparison of the box plots and data point distributions in each group in Fig. 4a reveals that none of the manipulated cockroaches venture as “far” on the trackball as one quarter of the animals in the “unmanipulated” group.

**Table 1:** Velocities and distances walked by the cockroach during the rotating stimulus at 500 lx

	Unmanipulated	Ocelli covered	Compound eyes covered	All eyes covered
Max. velocity (°/s) median	336.5	215.7	155.3	159.6
Mean ± SD	669.9 ± 703.5	241.3 ± 197.0	161.1 ± 105.3	156.9 ± 99.8
Average velocity (°/s) median	50.7	30.7	25.8	22.0
Mean ± SD	109.3 ± 138.1	33.8 ± 32.1	23.5 ± 19.0	23.2 ± 17.4
Total distance (°/30 s) median	1520.9	922.7	773.5	660.5
Mean ± SD	3278.1 ± 4141.7	1015.1 ± 962.4	706.4 ± 569.3	695.6 ± 520.6
Full rotations (#/30 s) median	4.2	2.6	2.1	1.9
Mean ± SD	9.1 ± 11.5	2.8 ± 2.7	2.0 ± 1.6	1.9 ± 1.4

Sample sizes are *n* = 920 (unmanipulated); *n* = 360 (Ocelli and compound eyes covered), *n* = 342 (all eyes covered)

### Walking velocities

Figure 4b compares the average angular walking velocities of the four treatment groups at different light intensities. Data obtained with all nine different stimulus frequencies are combined in each data point, so the plot reflects the general effect of light intensity on the walking activity of the animals. The differences in walking speeds between the treatments across light intensities stay similar to the differences seen in total distances at 500 lx (Fig. 4a), i.e., the velocity reading is always highest in the “unmanipulated” group and low intermediate in the “ocelli covered” group. The data points for “compound eyes covered” and “all eyes covered” groups are closely together, but statistically, the values are significantly different from each other at light intensities between 0.005 and 5 lx (two-tailed two-sample Kolmogorov–Smirnov test *p* < 0.05). At 500 lx, the values of the two groups are nearly identical.

To test whether the velocities of “compound eyes covered” and “all eyes covered” groups stay the same across all light intensities, data values of each group at different light intensities are compared. In both groups, the average velocities are similar at 0.005 and 0.05 lx (*p* > 0.40). In the “compound eyes covered”, group values of 0.05 and 5 lx do not differ from each other (*p* = 0.17), but there is a difference in “all eyes covered” group (*p* = 0.009). In both groups, the value at 500 lx is significantly different from the 5 lx value (*p* < 2.5 × 10^− 8^). Therefore, we conclude that the “compound eyes covered” and “all eyes covered” groups behave very similarly: at light intensities between 0.005 and 5 lx, the cockroaches in these groups walk at a fairly constant angular speed of about 15°/s, and at 500 lx, the walking speed increases to around 23°/s.

A remarkable observation in Fig. 4b is that the average walking velocity in the “unmanipulated” group never drops as low as it does in the three other groups, even when two data points (*n* = 216) at light intensities below the behavioural threshold are added to it. In the “unmanipulated” group, all other data points are significantly different from their neighbouring points (*p* < 0.01) except the two at the lowest intensities of 0.0005 and 0.0025 lx (*p* = 0.09), so the walking activity can be seen to reach a baseline of about 35°/s at light intensities below the behavioural threshold of 0.005 lx. Clearly, the eye manipulations affect the general walking activity of the cockroaches in the VR environment. The effect does not seem to be caused by the light deprivation alone, because the low-light ends of all the plots in Fig. 4b do not converge. The manipulation itself (paint or aluminium foil) seems to have an effect.

**Fig. 5:**
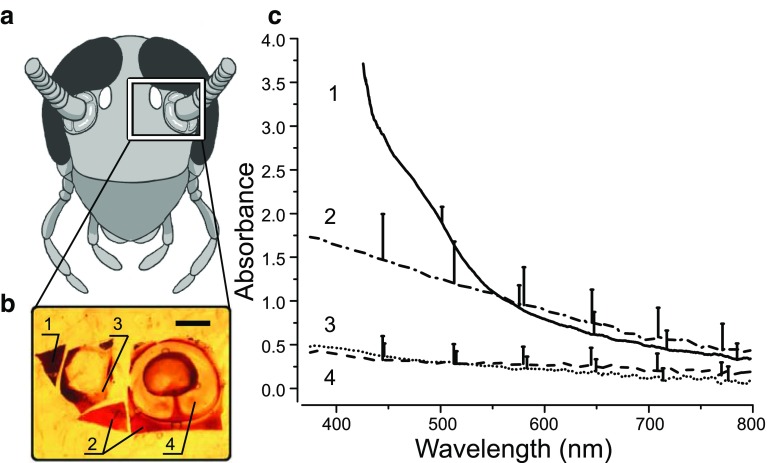
Absorbances of cuticle and antennal structures in the cockroach head capsule near the visual sense organs. **a** Schematic view of the cockroach head capsule. **b** Sample pieces used for the spectral absorbance measurements were chosen: 1: between the ocelli, 2: underside of the ocellus and antennal joint, 3: between the ocellus and antenna, and 4: the antenna base. Scale bar, 500 µm. **c** Averaged absorbance spectra ± SD from the positions numbered in **b** of the cockroach head. Sample sizes were: *N* = 3 animals, *n* = 5 measurements (sample 1); *N* = 3, *n* = 10 (2); *N* = 3, *n* = 9 (3); *N* = 3, *n* = 11 (4)

To explain the effect of the 500 lx intensity on the walking velocities of the “compound eyes covered” and “all eyes covered” groups (Fig. 4b), we measured the absorbance spectra of four different areas around the ocellus: the antennal socket, the light-brown cuticle near the antennal socket, the cuticle between the ocellus and the antenna, and the darkest cuticle around the ocellus (Fig. 5a, b). The measurements showed that these areas let up to 51% of the incoming light penetrate through into the subcutaneous tissue adjacent to the ocellar photoreceptors, and likely some of this light will excite the photoreceptors even when the ocellus itself is occluded (Fig. 5c).

Table 1 compiles cockroach activity data with different manipulations at 500 lx intensity and compares the measures of central tendency between the four groups. Maximum angular walking velocities are the transient top speeds of the animals. They behave similarly as the average angular velocities and total angular distances (Fig. 4): The central tendencies and dispersions are always highest in the “unmanipulated” group, intermediate in the “ocelli” group, and lowest in the “compound eyes” and “all eyes” groups. Total distances are also given in Table 1 as full rotations of the trackball during the 30-s rotation of the stimulus. These values show that even when all eyes are covered and the cockroaches are effectively in total darkness, on average, they still move more than the one full rotation required for reliable analysis of the trackball data (Takalo et al. 2012).

To analyse the movement activity further, percentage of the trials during which the cockroaches moved for less than one full rotation of the trackball during the stimulus movement (inactivity percentages) were calculated. Table 2 presents these data. In the “unmanipulated” group, the percentage of the low-activity trials at the four highest light levels stays fairly constant between 19.1 and 28.9% (mean 23.3%). Crossing of the behavioural threshold from 0.005 to 0.0025 lx is seen as a steep rise to 40.3% in the percentage of the low-activity trials. All the three manipulated groups have a slightly larger percentage to start with (mean 31.6%), and in the “ocelli” and “compound eyes” groups, it increases as the light level drops to 5 lx. At 0.005 lx in both the “ocelli” and the “compound eyes” groups in more than half of the trials cockroaches move for less than one full rotation in 30 s. In the “all eyes” group, the percentage does not rise above 44.4%, i.e., at intensities of 0.05 lx and below, it stays within the same range as in the “unmanipulated” group at and below 0.0025 lx.

**Table 2:** Percentages of trials with less than one full rotation travelled in 30 s at different light levels

	Unmanipulated	Ocelli covered	Compound eyes covered	All eyes covered
500 lx	20.3	30.6	34.4	29.8
5 lx	28.9	41.7	53.1	26.7
0.05 lx	19.1	41.9	51.4	44.4
0.005 lx	24.8	52.2	53.9	36.7
0.0025 lx	40.3			
0.0005 lx	44.9			

Sample sizes are *n* = 216–920 (unmanipulated); *n* = 360 (Ocelli and compound eyes covered), *n* = 90–342 (all eyes covered)

### Cuticular transmission

Spectral absorbance measurements show that light “leakage” occurs through the cockroach head capsule. Figure 5c illustrates the absorbances from the numbered locations (Fig. 5b) of the cuticle and antenna structures near the visual sense organs. For each spectrum, absorbance in the short-wavelength end is dominating. While absorbances are relatively high in samples 1 and 2, samples 3 and 4 are almost identical and transmit approximately half of all the incident light across the visible range. At the spectral sensitivity maximum of the ocellar green photoreceptor, at 500 nm (Goldsmith and Ruck 1958), absorbances are 1.97, 1.26, 0.30, and 0.29 in the order of numbering (corresponding to transmittances 1.1, 5.5, 50 and 51%), meaning that in the most translucent samples 3 and 4, approximately one in two incident photons could, in theory, be captured by the detector. Due to low light intensity on the specimen level and a high optical density in part of the samples, decreasing signal-to-noise ratio drastically, the spectra were truncated abruptly in the short-wavelength end.

## Discussion

### Ocellar stimulation modulates compound eye-mediated optomotor response

The response strengths in Figs. 2 and 3 confirm that the optomotor response is mediated by the compound eyes. Ocelli alone are not sufficient for the initiation of the response (Fig. 3a). However, there seems to be a strong facilitating effect of the ocelli on the optomotor response and the general activity level of the cockroaches (Fig. 4b). This is in accordance with the suggestion (e.g. Mizunami 1995a, d; Okada and Toh 1998) that the ocelli of the American cockroach could act as intensity measuring devices modifying the output of the compound eyes to adapt the responses to each ambient light condition.

In our previous experiments, we found that the visual system of the American cockroach is able to perceive moving stimuli and initiate appropriate motor responses down to the light level of 0.005 lx (Honkanen et al. 2014). Here, we report that when the ocelli have been covered but the compound eyes left intact, the optomotor response stops at a 1 decade higher intensity of 0.05 lx (Fig. 2g). In addition, the optomotor performance of cockroaches with covered ocelli seems to be worse at higher intensities than that of the intact animals, suggesting that ocellar stimulation strengthens or sensitises the responses by about 1.5–2 decades. Intracellular recordings from the ocellar L-neurons of the American cockroach have demonstrated a hyperpolarising on-response and depolarising off-spikes in response to white light stimuli down to light intensity of 0.003 lx. No responses were found in one decade dimmer light intensity of 0.0003 lx (Mizunami et al. 1982). These intensities correspond well with the results from our intracellular recordings from the photoreceptors of the cockroach compound eyes: quantum bumps were still seen at 0.005 lx but not at 0.0005 lx (Honkanen et al. 2014). Since the ocelli alone do not mediate the optomotor response, the behavioural results presented here indicate that when the ocelli are covered, the movement detection mechanism of the visual system, guided by the input from the compound eyes, stops responding to moving gratings at a clearly higher light intensity than with unhindered ocellar input. This may be the signal for the cockroach to switch from visually guided behavioural control to fully non-visual means when the ambient light gets too dim to produce reliable visual signals.

It seems that when the ocelli are covered (Fig. 2e–h) and thus signal to the animal a lower ambient light intensity than the compound eyes, the compound eye-initiated optomotor responses get slower (the cockroach becomes unable to react to the fastest stimulus frequencies) and coarser (unable to react to the slowest stimulus frequencies), than they would if the conditions were actually dim. This suggests that the ocelli, indeed, measure the average light intensity and that input from the ocelli modulates the signal from the compound eyes accordingly, causing increasing spatial and temporal summation of the signals. By comparing the compound eye ERG with and without ocellar occlusion in crickets, Rence et al. (1988) also came to the conclusion that the ocelli modulate the light intensity perception of the compound eyes. In addition, moths use their ocelli to measure ambient light intensity for photoentrainment, although there is no direct evidence for any ocellar input onto the compound eyes (Eaton et al. 1983; Wunderer and De Kramer 1989).

### Possible sites of ocellar modulation

In the American cockroach, the output from the ocelli goes to the optic lobes but does not reach the photoreceptors. On the basis of the extensive body of work on the ocelli of the American cockroach done by Mizunami and co-workers, it is known that all the L-neurons terminate in the ocellar tract neuropil where they synapse with the third-order neurons. Ocellar projections to all three neuropils of the optic lobes have been identified in the cockroach: six different types of third-order neurons connect to lobula and/or medulla either uni- or bilaterally (Mizunami and Tateda 1986; Mizunami 1995d), and a fourth-order neuron PS-LA1 from the posterior slope projects into the lamina (Mizunami 1995c). Similar connections to the lobula and the lamina as well as the same role of the ocelli in controlling the sensitivity of the compound eyes are found in the crickets, and these connections enable direct modulation of compound eye electroretinogram (ERG) by the ocelli (Rence et al. 1988). Small multimodal interneurons in the ocellar nerve of the cockroach respond, among other things, to compound eye stimulation (Ohyama and Toh 1986). Hence, there appears to exist the neural substrate needed for crosstalk between the ocelli and compound eyes.

There are several possible synaptic targets for ocellar inputs in the optic lobes. Cells in the lamina are indicated responsible of spatial summation in hawkmoths and the nocturnal bee Megalopta, and the same seems credible in the cockroach (Ernst and Fuller 1987; Ribi 1977; Stöckl et al. 2016; Theobald et al. 2006; Warrant et al. 2004), suggesting them as one plausible candidate for the ocellar input site affecting compound eye sensitivity. Ocellar projections to the lamina could even directly modulate the responses of the compound eye photoreceptors, as they do in crickets (Rence et al. 1988). The elementary movement detectors, responsible for detecting moving light and dark edges, of fly brains are located in the medulla (for review, see Behnia and Desplan 2015; Borst 2014; Silies et al. 2014). The “optomotor neurons” are found in the lobula complex (e.g., Borst 2014; Lillywhite and Dvorak 1981), so the ocellar projections there could possibly modulate the reaction directly. The motion-sensitive lobula plate tangential cell V1 in the blowfly responds to ocellar stimulation and seems a plausible site of combining signals from the two systems (Parsons et al. 2006). In addition, the descending contralateral movement detector (DCMD) in the ventral nerve cord of the locust responds to stimulation of the median ocellus (Simmons 1981). So far, there is no real evidence for or against any of these proposed sites in the cockroach.

### General activity level of the cockroach is also controlled by ocellar inputs

The general activity level of the cockroaches was studied here as reported in Fig. 4 and Tables 1 and 2. In Fig. 4a and Table 1, we compare the velocities and total distances walked by the unmanipulated animals and the three different manipulation groups at light intensity of 500 lx. The velocities and distances of the cockroaches that could not use their ocelli were always intermediate between the unmanipulated animals and those that could not use their compound eyes or any of their eyes. The decrease in the general activity levels in the manipulated groups can be seen as a decrease in the measures of central tendency but also as smaller dispersion in the data (compare the locations of the *X*’s marking the maximum values in Fig. 4a): cockroaches move less in general and there are no individuals that walk very long distances or at very high speeds when some or all of their eyes have been covered.

The average angular walking velocities of cockroaches with different eye manipulations at different light intensities are shown in Fig. 4b. The four manipulation groups behave in three distinct ways: in the “unmanipulated” group, where the cockroaches were able to use all of their four eyes, the walking speed increases rapidly with brightening light intensity. A similar but more modest increase is seen in the “ocelli covered” group where the cockroaches could use their compound eyes but not their ocelli to see the stimulus. The last two manipulations where the animals could use only their ocelli to see the stimulus (“compound eyes covered”) or where all the four eyes had been covered form the third distinct group and set a baseline of activity in these experiments.

The effect of covering the ocelli on the average walking velocities is substantial. The optomotor reaction is clearly mediated by the compound eyes, as shown in Figs. 2 and 3, but the general walking activity of the animals is dramatically reduced when they can use the compound eyes only (Fig. 4b). This could support the unproven idea of the ocelli as general stimulatory organs (e.g., Mizunami 1995b), but, perhaps, a simpler explanation would be negative phototaxis or photokinesis (e.g., Kelly and Mote 1990; Lazzari et al. 1998): when the ocelli do not signal bright light, cockroaches do not have the exigency to find a dark shelter, so they decrease their walking activity on the trackball. In contrast, ocellar occlusion does not decrease locomotor activity in the tiger moth (Wunderer and De Kramer 1989) or *Cataglyphis* ants, but the walking velocity of the ant is markedly reduced when the compound eyes are occluded (Fent and Wehner 1985).

The head cuticle of the American cockroach is brownish in colour and, in bright light such as under a microscope, quite translucent. Weber and Renner (1976) note that the ocelli of the American cockroach lack all structures associated with optical isolation except for a tapetum, which envelopes the proximal ends of photoreceptors across one quarter of the ocellus. In the light of the general activity levels of the cockroaches, it seems possible that some light could reach the photoreceptors of the ocelli diffusely through the head capsule, as also suggested by Roberts (1965), Eaton (1971), and Okada and Toh (1998). We tested this by measuring the absorbance spectra of the antennal socket and three different areas of the head cuticle around the ocellus (Fig. 5). At the maximally exciting wavelength for ocellar photoreceptors, 500 nm (Goldsmith and Ruck 1958), the cuticle acts as a 0.3–2.0 decade filter, i.e., a fraction (51–1.1%) of the incoming light does get through it. The little tissue in between the cuticle around the ocellus and the ocellar photoreceptors is light-coloured and should not attenuate the incoming light very much. The tapetum covers only one-fourth of the area of the ocellar cup (Weber and Renner 1976), and, therefore, forms only partial isolation from light entering from the side. Considering the high sensitivity of cockroach photoreceptors (Heimonen et al. 2006, 2012; Honkanen et al. 2014) and the stimulating effect of activation in the green-sensitive photoreceptors of the cockroach (Zhukovskaya et al. 2017), it seems likely that this light on the ocellar photoreceptors is enough to affect the activity of the animal. The translucent head capsule can explain the higher average walking velocities of the cockroaches in the “compound eyes covered” and “all eyes covered” groups at 500 lx in comparison to the lower light intensities.

The general movement activity of the cockroaches is analysed further in Table 2 as the percentage of trials during which the animals moved for less than one full rotation of the trackball (inactivity percentage). Various periods of inactivity interspersed between bouts of walking are a typical feature of cockroach behaviour in experimental settings (Takalo et al. 2012; Zhukovskaya et al. 2017). In the “unmanipulated” group, the crossing of the behavioural threshold (Honkanen et al. 2014) increases the percentage steeply from ca. 23% in the four highest intensities to ca. 43% at the two lowest intensities. This can be interpreted, so that the negatively phototactic cockroaches are more likely to allocate time for other functions than locomotion when they do not sense the presence of light. At the same time, however, there remains some walking activity, which is probably due to the inability of the animals to make an antennal contact with any solid object when they are walking on the trackball (Okada and Toh 2000).

In all the manipulated groups, the inactivity percentage at 500 lx is a bit higher than in the “unmanipulated” group, around 30%. In the “compound eyes covered” group where the animals could only “see” with their ocelli, a threshold is seen already between 500 and 5 lx intensities. As Fig. 4b shows in all of the manipulated groups, 500 lx causes a higher walking velocity than the other intensities. The 500 lx intensity seems to be bright enough to cause activity irrespective of the manipulation. For a cockroach that cannot find with its antennae any solid objects to hide under it is imperative that it gets out of bright, open spaces where it could be seen by a predator (Okada and Toh 2000). It seems that 500 lx is bright enough, so that this effect is caused even by light entering the photoreceptors through only the head capsule (“all eyes covered” group; see also Fig. 5). At intensities of 5 lx and below, more than half of the trials with cockroaches that could use only their ocelli yielded a total walking distance of less than one full rotation of the trackball. The animals that could only use their compound eyes (“ocelli covered” group) seem to have two thresholds in the inactivity data. The higher one is between the same intensities as in the “compound eyes covered”. The lower one between 0.05 and 0.005 lx corresponds to the optomotor behavioural threshold with this manipulation (Fig. 2g, h). Below that the inactivity percentage increases to 52.2%.

Surprisingly, the inactivity percentage of the group where all four eyes had been covered never reached 50%. In fact, the inactivity values at 0.05 and 0.005 lx resemble those of the “unmanipulated” group at and below 0.0025 lx. What causes the higher inactivity percentages in the “ocelli covered” and “compound eyes covered” groups? The idleness could be caused by the conflicting signals from the compound eyes and the ocelli. When both systems signal a similar light intensity, as in the “unmanipulated” and “all eyes covered” groups, cockroaches maintain a higher basic activity (although not necessarily higher velocity) on the trackball. Possibly, the conflicting signals make the cockroaches walk more slowly and stop more often to collect more light to make sense of the input, as animals often compensate for their slower and coarser vision in darkness by moving more slowly (e.g., Narendra et al. 2013; Theobald et al. 2007). The animals may also be spending more time cleaning the pair of eyes that are signalling for a lower intensity.

### Conclusions and future prospects

The optomotor reaction is mediated by the compound eyes and cannot be elicited with stimulation via the ocelli alone. Input from the ocelli improves the sensitivity of the movement detection system in dim light and enhances the general walking activity of the cockroaches in comparison to the manipulation in which the ocelli could not be used. The compound eye photoreceptors seem to be responsible for the general movement activity and the ocelli alone are not sufficient for causing a higher basal activity. The basal walking activity of the unmanipulated cockroaches is always higher than in any of the manipulation groups, indicating that the manipulations themselves affect the walking activity, possibly by adding extra weight to the heads of the cockroaches. Our findings call for a further investigation of the ocellar inputs in the compound eyes and optic lobes of the American cockroach to determine the mechanism by which ocelli modulate the optomotor response. The first step would be simultaneous ocellar manipulations and either intracellular recordings coupled with dye injections or ERG recordings of the compound eyes to determine whether a direct modulation of the compound eye photoreceptors exists, followed by recordings in the optic lobes if necessary.
